# Climbing Fibers Provide Graded Error Signals in Cerebellar Learning

**DOI:** 10.3389/fnsys.2019.00046

**Published:** 2019-09-11

**Authors:** Yunliang Zang, Erik De Schutter

**Affiliations:** Computational Neuroscience Unit, Okinawa Institute of Science and Technology Graduate University, Okinawa, Japan

**Keywords:** cerebellar learning, Purkinje cell, climbing fiber, complex spike (CS), error signal

## Abstract

The cerebellum plays a critical role in coordinating and learning complex movements. Although its importance has been well recognized, the mechanisms of learning remain hotly debated. According to the classical cerebellar learning theory, depression of parallel fiber synapses instructed by error signals from climbing fibers, drives cerebellar learning. The uniqueness of long-term depression (LTD) in cerebellar learning has been challenged by evidence showing multi-site synaptic plasticity. In Purkinje cells, long-term potentiation (LTP) of parallel fiber synapses is now well established and it can be achieved with or without climbing fiber signals, making the role of climbing fiber input more puzzling. The central question is how individual Purkinje cells extract global errors based on climbing fiber input. Previous data seemed to demonstrate that climbing fibers are inefficient instructors, because they were thought to carry “binary” error signals to individual Purkinje cells, which significantly constrains the efficiency of cerebellar learning in several regards. In recent years, new evidence has challenged the traditional view of “binary” climbing fiber responses, suggesting that climbing fibers can provide graded information to efficiently instruct individual Purkinje cells to learn. Here we review recent experimental and theoretical progress regarding modulated climbing fiber responses in Purkinje cells. Analog error signals are generated by the interaction of varying climbing fibers inputs with simultaneous other synaptic input and with firing states of targeted Purkinje cells. Accordingly, the calcium signals which trigger synaptic plasticity can be graded in both amplitude and spatial range to affect the learning rate and even learning direction. We briefly discuss how these new findings complement the learning theory and help to further our understanding of how the cerebellum works.

## Introduction

It is widely recognized that the cerebellum is critical in coordinating muscles and learning novel movements with highly accurate and temporal precision. Even for a simple finger-to-nose task, to make different segments of hand and arm interact smoothly, humans need the cerebellum to precisely modulate the sequence and duration of elementary movements. The cerebellum has a relatively simple anatomy and the anatomic connections involved in its associated functions are also well known. In this context, the cerebellum becomes an ideal structure to explore learning rules and it also opens a window for us to begin to comprehend how the brain works.

**Figure 1:**
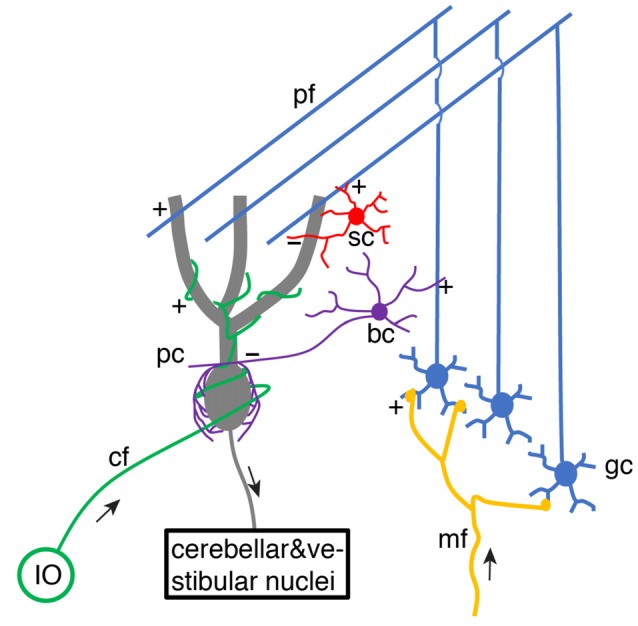
Schematic of basic cerebellar circuitry. mf, gc, pf, pc, sc, bc, cf, IO designate mossy fiber, granule cell, parallel fiber, Purkinje cell, stellate cell, basket cell, climbing fiber and inferior olive neuron, respectively. “+” and “−” correspond to excitatory and inhibitory synaptic connections, respectively. pf → pc and pf → bc/sc → pc connections form a typical feed-forward-inhibition circuit. Some cell types and connections have been omitted for simplicity (Schematic, created by ourselves).

For decades, it has been of great interest to decipher cerebellar learning algorithms (De Schutter, 1995). The cerebellar learning theory was first systematically proposed by Marr (1969) and then Albus (1971), building upon previous knowledge of wiring connections and electrophysiological properties of the cerebellar cortex (Eccles et al., 1967). The basic structure of cerebellar circuitry is illustrated in Figure 1. Mossy fibers transmit sensory and cortical information to granule cells *via* excitatory synaptic connections. Small granule cells are electrically compact and they constitute the majority of neurons in the brain. Their axons project up into the molecular layer of the cerebellar cortex, bifurcate and form excitatory synapses onto Purkinje cell dendrites. As the sole output of the cerebellar cortex, each Purkinje cell is contacted by ~150,000 parallel fiber (bifurcations of granule cell axons) synapses. Meanwhile, parallel fibers also activate stellate cells and basket cells, which form inhibitory synapses with Purkinje cells, establishing a stereotypical feed-forward-inhibition circuit. Stellate cells tend to locate in the outer part of the molecular layer and mainly target Purkinje cell distal dendrites. In contrast, basket cells locate in the inner part of the molecular layer and mainly target Purkinje cell somas and axon initial segments (AIS).

Each Purkinje cell is also innervated by a single climbing fiber, which spontaneously fires at ~1 Hz and reliably triggers complex spikes. In Marr’s theory (Marr, 1969), when a novel movement needs to be learned or an old one requires modification because of an error, the climbing fiber fires a spike. Then the simultaneously activated parallel fiber synapses in Purkinje cells are potentiated to learn. Albus extended Marr’s model and proposed that synaptic weights between parallel fibers and Purkinje cells should be depressed rather than potentiated since Purkinje cells are inhibitory neurons (Albus, 1971). Amazingly, the idea of climbing fiber-induced plasticity at parallel fiber synapses being the cellular substrate of cerebellar learning was proposed before any experimental demonstration of climbing fiber-evoked parallel fiber plasticity. Ito and Kano (1982) found that the synapses between parallel fibers and Purkinje cells undergo long-term depression (LTD) when parallel fibers are activated in conjunction with climbing fibers. Since then, the Marr-Albus-Ito theory has dominated cerebellar learning research, although it has been challenged by emerging experimental data at molecular, cellular, and behavioral levels in recent years. First, this theory turns out to be incomplete after the discovery of long-term potentiation (LTP) at parallel fiber-Purkinje cell synapses (Sakurai, 1987) and synaptic plasticity at other sites. In the cerebellar cortex, mossy fiber-granule cell synapses, granule cell-Golgi cell synapses, Golgi cell-granule cell synapses, parallel fiber-molecular layer interneuron (MLI) synapses, and MLI-Purkinje cell synapses are all plastic (Gao et al., 2012). Mossy fiber-cerebellar nuclei neuron synapses can also be potentiated (Pugh and Raman, 2006). In theory, all these learning sites should work synergistically to optimize motor behaviors. Second, climbing fibers seemed to be inefficient teachers in cerebellar learning. Since multi-site learning was well summarized by Gao et al. (2012), we will review the history of climbing fiber physiology, summarize recent progress, and predict how climbing fibers may instruct cerebellar learning.

##  “All-or-None” Climbing Fiber Responses

**Figure 2:**
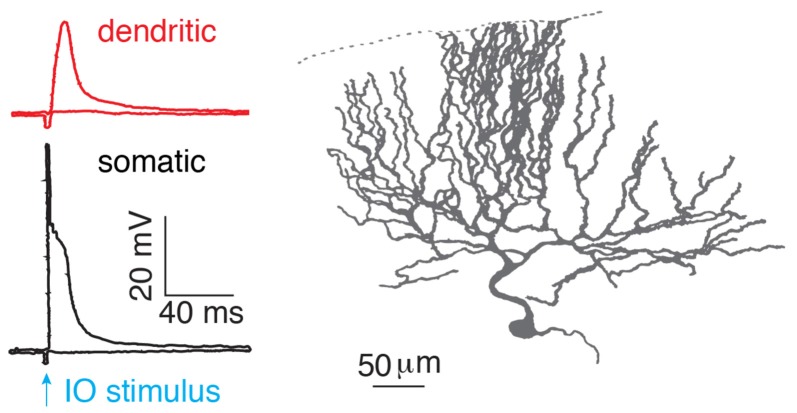
“All-or-none” climbing fiber responses. Climbing fiber-evoked somatic (black) and dendritic (red) responses measured in the isolated cerebellum of turtles. The climbing fiber was activated by stimulating an inferior olive neuron (IO), reproduced from Hounsgaard and Midtgaard (1989). Somatic complex spikes are characterized by a fast spike followed by several spikelets on top of a plateau potential. © 1989 The Physiological Society, reproduced with permission from Wiley Publishing, Inc.

After birth, each Purkinje cell is initially innervated by several climbing fibers (Hashimoto and Kano, 2003). With development, only one of the climbing fibers is selectively strengthened and preserved, while the weaker ones are eliminated. Thus, in the adult cerebellar cortex, preserved climbing fibers provide powerful synaptic inputs to Purkinje cells, with ~300 synapses distributed on the soma and proximal parts of each dendritic tree (Llinas et al., 1969). More than 50 years ago, the climbing fiber response in Purkinje cells was described as “all-or-none” by Eccles et al. (1966). In response to a climbing fiber stimulus, stereotypical complex spikes occur at the soma with an initial fast spike and several spikelets driving on a plateau membrane potential (Eccles et al., 1966; Hounsgaard and Midtgaard, 1989; Figure 2). In the dendrite, the climbing fiber-evoked response is distinct from the somatic complex spike. The dendritic response was also found to be “all-or-none” (Llinás and Sugimori, 1980; Hounsgaard and Midtgaard, 1989), which corresponded to either global Ca^2+^ influx in the whole dendrite, triggered by a strong stimulus, or no Ca^2+^ influx from a weak, subthreshold stimulus (Miyakawa et al., 1992). The view of “all-or-none” climbing fiber responses in Purkinje cells continues to be emphasized in recent cerebellar research (Piochon et al., 2007; Nietz et al., 2017; Bouvier et al., 2018).

Unfortunately, “all-or-none” responses make the climbing fiber an inefficient teacher and this view has triggered heated debates regarding its functional importance in cerebellar learning (Najafi and Medina, 2013). First, spontaneously firing climbing fibers provide “instruction” signals, even in the absence of errors, causing a signal-to-noise problem that is inherent in any spontaneously active system (Llinás et al., 1997). According to cell-attached recordings, granule cells fire at 4.8 ± 1.3 Hz under resting condition in awake mice (Chen et al., 2017). Thus, parallel fiber synapses coincident with spontaneous complex spikes become modified, since single Purkinje cells would seem unlikely to differentiate spontaneous “noise” complex spikes from “signal” complex spikes if their responses were “all-or-none.” Second, unlike simple spikes, the firing rate of climbing fibers is low, implying that their capacity to increase the information transmission by changing spiking rates is limited (Kitazawa et al., 1998). Third, the plasticity of parallel fiber synapses occurs on Purkinje cell dendrites. “All-or-none” dendritic responses suggest that climbing fibers carry only qualitative rather than quantitative information, which significantly constrains the information capacity of climbing fibers and the learning capacity of single Purkinje cells. Finally, cerebellar learning suffers from the credit assignment problem (Minsky, 1961; Suvrathan et al., 2016; Bouvier et al., 2018). To learn well-timed and precise arbitrary movements, individual Purkinje cells most probably require specific error signals and learn heterogeneously. However, “all-or-none” climbing fiber responses would make individual Purkinje cells unable to extract specific error information from global error feedback.

## Graded Climbing Fiber Responses in Purkinje Cells

It is necessary to reassess a neglected aspect of experimental data reported in the last century that seemed to support “all-or-none” climbing fiber responses. Although neither climbing fiber-evoked somatic complex spikes nor dendritic spikes change by varying stimulus amplitude in the same cells *in vitro*, their firing patterns vary significantly among different cells and between recordings by different groups (Eccles et al., 1966; Llinás and Sugimori, 1980; Hounsgaard and Midtgaard, 1989). Observed complex spikes *in vivo* also show quite variable firing patterns (Bell and Kawasaki, 1972; Gilbert, 1976; Armstrong and Rawson, 1979). Theoretically, several factors can potentially reconcile these observations. Climbing fiber responses in Purkinje cells exhibit individual variability. The characteristics of Purkinje cells, such as excitability or voltage, varies. Presynaptic climbing fiber input varies, although Crill (1970) contested this. Do these factors really occur and grade climbing fiber responses?

**Figure 3:**
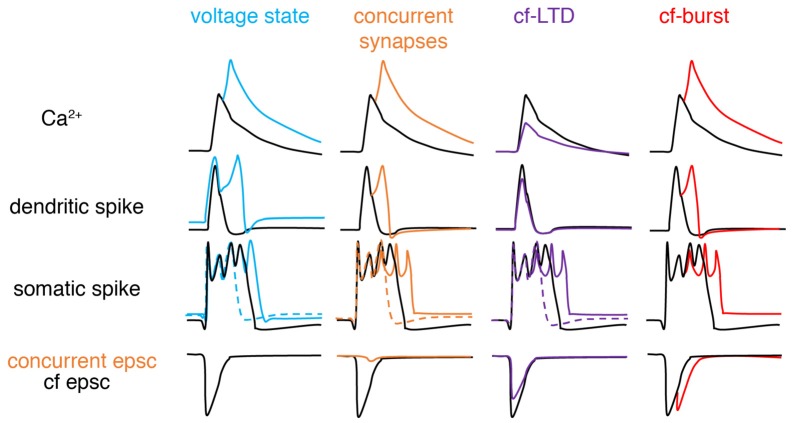
Schematic of factors modifying climbing fiber responses. From left to right, climbing fiber (cf) responses can be graded by voltage states, concurrent synaptic input, cf-long-term depression (LTD), and spike numbers in a cf-burst. Compared with basal conditions (black): depolarization (blue), concurrent excitatory synapse (orange), cf-LTD (purple) and cf-burst (red, manifested by cf epsc) increases, increases, decreases and increases dendritic Ca^2+^ influx respectively, by modulating dendritic spikes. If concurrent synaptic input is inhibitory, changes are opposite (not illustrated here). For somatic complex spike changes, the existence of both dashed and solid-colored traces suggests that complex spikes can exhibit bidirectional changes depending on the “state,” also implying that somatic complex spikes are poor proxies for dendritic responses. Sketched according to Zang et al. (2018; the authors’ open access paper).

In recent years, the veil obscuring the complex nature of climbing fiber responses has gradually lifted (Figure 3). The somatic complex spike is voltage-dependent and shows significant individual variability (Khaliq and Raman, 2005; Monsivais et al., 2005). Later, Tal et al. (2008) and Rokni et al. (2009) found that apart from somatic complex spike patterns, dendritic Ca^2+^ influx also shows strong voltage-dependence in slice preparations, characteristic of controversial bistability (Loewenstein et al., 2005; Schonewille et al., 2006), and suggesting that climbing fiber responses may be ternary rather than binary. Similar findings were later confirmed in anesthetized rats *in vivo* (Kitamura and Hausser, 2011). Ionic current modulation is also shown to grade the amplitude and spikelet number of dendritic spikes (Ohtsuki et al., 2012; Otsu et al., 2014). Interestingly, although both somatic and dendritic responses undergo modulation, conflicting observations have been made regarding their interactions. The variation of dendritic spikes had only a minimal role in regulating somatic output in Callaway et al. (1995), Davie et al. (2008) and Rowan et al. (2018), but not in Ohtsuki et al. (2012) or Otsu et al. (2014). The climbing fiber-Purkinje cell synapses can also undergo LTD, which modulates complex spike waveforms by reduced synaptic current (Hansel and Linden, 2000). The capability of climbing fibers in generating analog signals was further demonstrated by Mathy et al. (2009). Depending on the phase of subthreshold oscillations, single somatic action potentials in olivary neurons can be translated into bursts of varying numbers of axonal spikes in climbing fibers and then reliably conveyed to Purkinje cells. Increasing spike numbers in climbing fibers modulate somatic spike patterns, enhances dendritic spikes, and consequently promote short-term and long-term plasticity at parallel fiber synapses in Purkinje cells. More recently, Gaffield et al. (2019) demonstrated that climbing fiber burst firing occurs *in vivo* and this response can be modulated by behaviorally relevant stimuli. Importantly, Purkinje cell dendrites can integrate this burst firing into a graded Ca^2+^ response. In addition, complex-spike doublets have been also shown to increase Ca^2+^ influx compared with single complex spikes and have been suggested to be “instruction” signals compared to spontaneous “noise” single complex spikes (Titley et al., 2019).

**Figure 4:**
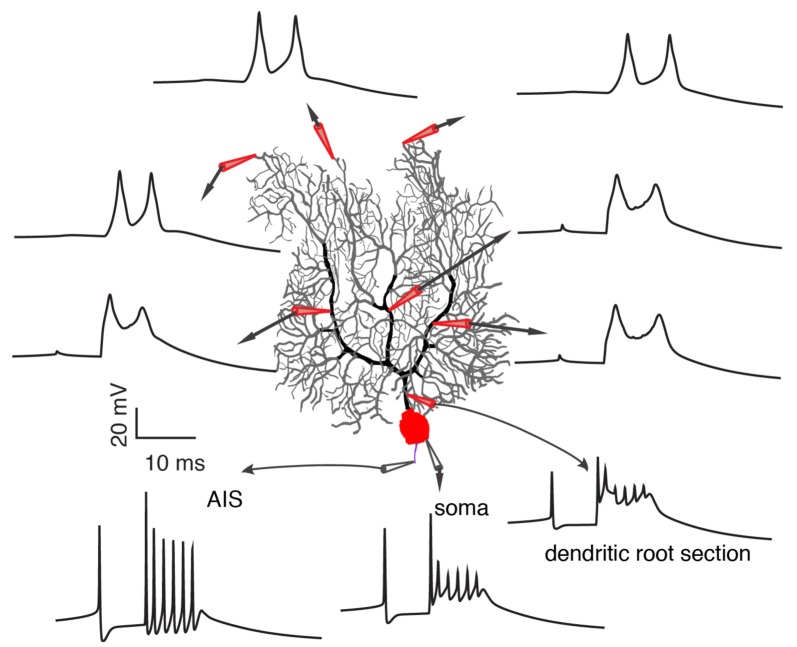
Occurrence of somatic and dendritic spikes. Climbing fiber responses at different sites of the Purkinje cell, including axon initial segment (AIS), soma, proximal and distal dendrites. Each spikelet in the complex spike still initiates at the AIS. Reproduced from Zang et al. (2018; the authors’ open access paper).

On one hand, a growing body of experimental evidence demonstrates the variability of climbing fiber responses, but on the other hand, it fails to provide a systematic explanation of somatic and dendritic spike initiation and variation and leads to many conflicting observations that stymie delineation of the functional role of climbing fibers in cerebellar learning. Furthermore, limited information extracted from noisy *in vivo* data by present experimental techniques (local field potential, calcium imaging, voltage imaging), adds to the confusion. In a recent experimental data-based theoretical study by Zang et al. (2018), the biophysical mechanism of climbing fiber responses at whole Purkinje cell scale was systematically investigated (Figure 4). The somatic complex spike is a result of climbing fiber synaptic current, intrinsic ionic currents, and axial currents from the dendritic spike. Accordingly, it is subject to regulation by climbing fiber firing patterns, voltage-dependent availability of Na^+^ channels, and dendritic spike patterns.

In agreement with Ohtsuki et al. (2012) and Otsu et al. (2014), dendritic spike patterns can modulate somatic spike outputs in the model. The possible reason that Davie et al. (2008) and Rowan et al. (2018) observed a minimal role of dendritic spikes in regulating somatic firing patterns is that dendritic spikes measured by single-site patch-clamps do not reliably represent dendritic spike patterns in the complete dendrite. Zang et al. (2018) reproduced the data of Davie et al. (2008) and showed that the somatic complex spike hardly changes when a dendritic spike is either completely local or propagates along a branch. On the dendritic side, Ca^2+^ influx can be graded by climbing fiber firing patterns, coincident background synapses, and voltage states in an analog manner. The conflicting spatial range of Ca^2+^ influx observed in different experiments can be accounted for by different voltage states (Miyakawa et al., 1992; Zagha et al., 2010; Kitamura and Hausser, 2011; Ohtsuki et al., 2012; Otsu et al., 2014). On distal parts of Purkinje dendrites, there are not only P-type voltage-dependent Ca^2+^ channels, but also voltage-dependent K^+^ channels. Climbing fibers directly excite and depolarize proximal smooth dendrites (Palay and Chan-Palay, 1974; Roth and Häusser, 2001; Zang et al., 2018), but not distal spiny parts. The axial current from the depolarization of proximal parts constitutes the sole current source to depolarize distal parts and to reach the activation threshold of P-type Ca^2+^ channels. When dendritic membrane potential is hyperpolarized, the large availability of K^+^ currents outweighs P-type Ca^2+^ current to “brake” the initiation of dendritic spikes in distal parts. With depolarization, K^+^ currents inactivate and axial currents can trigger dendritic spikes in the whole dendrite. Theoretically, voltage-dependent climbing fiber responses also endow single Purkinje cells with the ability to overcome the credit assignment problem. Individual Purkinje cells can extract specific instruction information according to their firing states (equivalent to voltage states *in vivo*; Jelitai et al., 2016), even when they receive the same climbing fiber signal.

## Graded Error Signals in Cerebellar Learning

Although Purkinje cells and climbing fibers are capable of encoding analog signals, does this really contribute to cerebellar learning? As reported by Najafi et al. (2014b), sensory event-triggered Ca^2+^ influx is larger than spontaneous Ca^2+^ spikes to signal the occurrence of an unexpected sensory event. Furthermore, Ca^2+^-based instruction signals in Purkinje cell dendrites contain analog information that encodes the strength of instructive stimuli trial-by-trial (Najafi et al., 2014a). In another study, magnitudes of both plasticity and motor learning are correlated with durations of complex spikes measured in monkeys executing eye pursuit movements (Yang and Lisberger, 2014). On one hand, this finding is ground breaking, since it demonstrated for the first time that climbing fibers can adjust the instruction signal according to real-time movement errors. However, the mechanism that realizes the analog instruction signal remains unknown and requires further work. The authors assumed that the analog instruction signal is encoded by the number of spikes in climbing fibers (Mathy et al., 2009), ignoring other factors contributing to complex spike duration, such as firing states and background synapses in Purkinje cells (Kitamura and Hausser, 2011; Rowan et al., 2018; Zang et al., 2018). On the other hand, it remains unclear whether somatic complex spikes are reliable proxies of dendritic Ca^2+^ influx in Purkinje cells. Extracellular microelectrodes are usually used to extract simple spikes and complex spikes *in vivo*. Nonetheless, complex spike patterns are extremely variable in spikelet numbers and durations (Warnaar et al., 2015; Tang et al., 2017), and cannot be well separated, in most cases. Purkinje cell simple spike firing rates show bidirectional modulation (i.e., increased or decreased) during tasks (Yang and Lisberger, 2014; Herzfeld et al., 2015; Chen et al., 2016; Jelitai et al., 2016). At high voltage states (high firing rate), amplitudes of spikelets in complex spikes tend to decrease (Warnaar et al., 2015; Zang et al., 2018) and they are prone to *in vivo* noise. Thus, it is easier to sort complex spikes from Purkinje cells with low firing rates compared to Purkinje cells with high firing rates, and this causes unavoidable bias in statistical analyses. Sometimes, it is even impossible to separate a complex spike with its subsequent simple spike, when the complex spike lacks a significant pause. This situation mainly occurs when there is only one dendritic spikelet that fails to hyperpolarize dendrites (see Figure 6A in Davie et al., 2008). Additionally, complex spike duration does not linearly correlate with dendritic Ca^2+^ influx (Zang et al., 2018), making the complex spike an unreliable proxy of dendritic response even if it can be well separated. Finally, and most importantly, the cellular mechanism of this short-term trial-by-trial learning is still unclear and how it correlates with long-term learning is unknown (Kimpo et al., 2014). Short-term plasticity of parallel fiber synapses and dendritic excitability plasticity may also be candidate mechanisms (Rancz and Häusser, 2006; Mathy et al., 2009; Ohtsuki et al., 2012; Regehr, 2012; Grangeray-Vilmint et al., 2018).

Noticeably, theoretical studies have also started to test the role of graded climbing fiber responses in cerebellar learning. Using a cerebellar network model with necessary climbing fiber-evoked burst-pause information in Purkinje cells (different with parallel fiber-evoked burst-pause by Steuber et al., 2007), Luque et al. (2019) found that parametric “pause” signals help to support both early and consolidated vestibulo-ocular reflex learning.

## LTD and LTP in Cerebellar Learning

**Figure 5:**
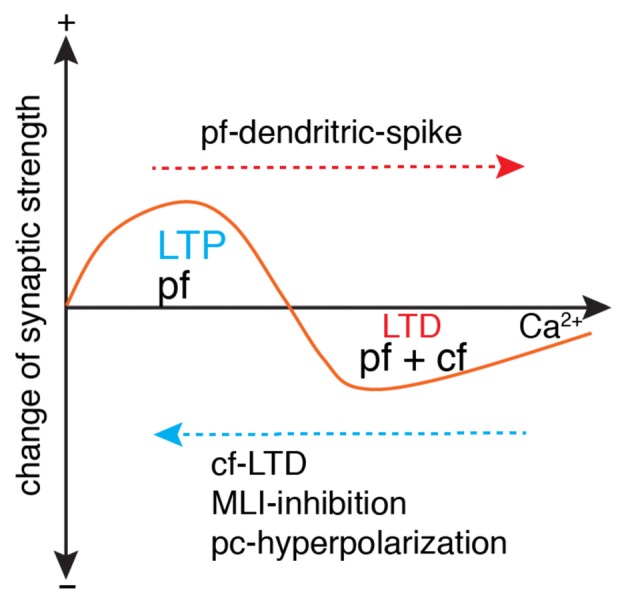
Ca^2+^-determined bidirectional plasticity of parallel fiber synapses. Parallel fiber (pf)-LTD has a higher Ca^2+^ induction threshold (usually induced by conjunctive pf and cf stimulation) than long-term potentiation (LTP; usually induced by pf stimulation in isolation). Depending on the dendritic Ca^2+^ influx, LTP can also be induced by conjunctive pf and cf activation under the condition of cf-LTD, conjunctive inhibition by molecular layer interneuron (MLI), or Purkinje cell (pc)-hyperpolarization. LTD can also be induced by pf in isolation when dendritic spikes occur with strong pf stimulation. Modified from Coesmans et al. (2004).

Parallel fiber synapses exhibit bidirectional long-term plasticity (Coesmans et al., 2004; Gallimore et al., 2018; Zamora Chimal and De Schutter, 2018), i.e., depression and potentiation. LTP is necessary to prevent suppression of all parallel fiber synapses due to spontaneous activation of parallel fibers and climbing fibers. Compared with LTP, LTD has a higher Ca^2+^ induction threshold, usually with a climbing fiber as the polarity switch (Figure 5). However, climbing fiber activation is neither sufficient nor necessary to trigger LTD, as evidenced by the LTP triggered by climbing fiber-LTD (Coesmans et al., 2004), and LTD triggered by strong parallel fiber stimulation alone (Hartell, 1996). Mathy et al. (2009) required an even higher threshold for LTD induction. Pairing of parallel fiber stimuli with a climbing fiber burst triggers LTD of parallel fiber synapses, but pairing of parallel fiber stimuli with a single climbing fiber stimulus induces LTP. Recently it was shown that the calcium threshold can slide rather than being constant (Piochon et al., 2016). Pooling the data together, it is easy to find that polarity of synaptic changes essentially depends on the amplitude of dendritic Ca^2+^ influx relative to the threshold, regardless of concurrent climbing fiber signals (Figure 5). In the Albus-Ito theory, LTD of parallel fiber synapses forms the unique cellular substrate of cerebellar learning, and LTP is largely ignored (Not by Marr, 1969). Nonetheless, whether LTD plays an essential role in cerebellar learning was questioned strongly in recent years. In mutant mice with deficits in parallel fiber-Purkinje cell LTD, there was no learning impairment in cerebellar coordination tasks, including adaptation of vestibulo-ocular reflex, eyeblink conditioning, and locomotion learning (Schonewille et al., 2011). The authors argued that LTD of parallel fiber synapses was not essential for cerebellar learning. However, Yamaguchi et al. (2016) demonstrated that LTD is inducible by intensified conjunctive stimulation in the same types of mutant mice, refuting previous arguments that questioned the function of LTD in cerebellar learning. Obviously, the lack of a standardized LTD stimulation protocol has caused many conflicting results (Suvrathan et al., 2016; Bouvier et al., 2018; Suvrathan and Raymond, 2018) and there is a lack of systematic experimental evaluation of the effect of stimulation parameters on the probability of plasticity induction (Zamora Chimal and De Schutter, 2018). In a more recent study, by developing a new optogenetic tool to transiently manipulate parallel fiber synaptic plasticity, Kakegawa et al. (2018) demonstrated that LTD is directly responsible for motor learning during horizontal optokinetic response and vestibulo-ocular reflex.

Although LTD is demonstrably critical in cerebellar learning, at least in some behavioral contexts, there is increasing awareness that it is not the sole mechanism. Accumulating evidence demonstrates that LTP of parallel fiber synapses also functions in procedural learning (Schonewille et al., 2011; Grasselli and Hansel, 2014; Gutierrez-Castellanos et al., 2017; Romano et al., 2018). Consistent with cerebellar plasticity rules in slice preparations [LTP usually induced by isolated parallel fiber activation (Coesmans et al., 2004)], potentiation of parallel fiber synapses is prominent in Purkinje cells with low complex activation probability (Romano et al., 2018). Interestingly in another study, optogenetic activation of molecular interneurons shifts the climbing fiber-induced depression of parallel fiber synapses to potentiation in vestibulo-ocular reflex learning (Rowan et al., 2018). This is the first evidence to support climbing fiber-triggered LTP during cerebellar learning, although abundant data support climbing fiber-induced LTP in slice preparations. It is mechanistically easy to understand that enhanced molecular interneuron spiking reduces climbing fiber-triggered dendritic Ca^2+^ influx and consequently shifts plasticity from LTD to LTP.

In cerebellum-related behaviors such as saccadic eye movement (Herzfeld et al., 2015; Hong et al., 2016), self-paced locomotion (Jelitai et al., 2016) and voluntary whisking (Chen et al., 2016), Purkinje cells show bidirectional modulation of their simple spike firing rates. Then a remaining, neglected, critical question is to measure whether climbing fiber-evoked Ca^2+^ influx is constrained to proximal dendrites in Purkinje cells showing decreased firing rates, as observed *in vitro* (Zagha et al., 2010; Otsu et al., 2014; Zang et al., 2018). Even if Ca^2+^ influx is still global in Purkinje cell dendrites, is it within the range of triggering LTP rather than LTD? In other words, does the directional change of Purkinje cell simple spike firing rates determine the polarity of parallel fiber synaptic strength changes, with LTD in “bursting” Purkinje cells, but LTP in “pause” Purkinje cells?

A recent study proposed synaptic plasticity rules that strikingly contradict the current consensus of plasticity induction to solve the credit assignment problem (Bouvier et al., 2018). In their new rule, parallel fiber stimulation requires both conjunctive perturbation (spontaneous) and post-erroneous climbing fiber signals to induce LTD. With only conjunctive perturbation or post-erroneous climbing fiber signals, parallel fiber synapses undergo LTP or no change. In theory, this new plasticity rule still works with the Ca^2+^ threshold-dependent plasticity rule (Coesmans et al., 2004), but obviously more experimental data are required to support this algorithm in motor learning, since existing oculomotor control data do not (Catz et al., 2005; Ke et al., 2009; Yang and Lisberger, 2014).

## Branch-Specific Dendritic Responses and Learning

Conventionally, dendritic trees are thought to just receive synaptic inputs from presynaptic cells and to convey them to the soma. In recent years, more and more experimental data have uncovered local computation in neuronal dendrites (Branco and Häusser, 2010; Cichon and Gan, 2015). In Purkinje cells, the parallel fiber synapses distributed on spiny dendrites undergo synaptic plasticity. The spatio-variability of dendritic Ca^2+^ influx determines the computational unit of climbing fiber responses and constrains the learning capacity of individual Purkinje cells. Although Ca^2+^ influx due to strong parallel fiber-triggered dendritic spikes is constrained to branchlets (Hartell, 1996; Rancz and Häusser, 2006), information about spatio-temporal patterns of climbing fiber-evoked dendritic responses has only become available in recent years. The amplitude of dendritic Ca^2+^ signals was first shown to increase with distance from the soma in anesthetized rats (Kitamura and Hausser, 2011). This can be explained either by increased surface-to-volume ratios or non-uniform distribution of Ca^2+^ channels with distance from the soma. In the physiologically detailed Purkinje cell model with homogeneous distributed Ca^2+^ channels, dendritic Ca^2+^ influx increases significantly with distance from the soma and shows significant variations in different branchlets at similar distances (Zang et al., 2018). As discussed above, the initiation of “out-of-territory” dendritic spikes on distal dendrites relies on the axial current from climbing fiber-depolarized proximal dendrites. The morphology-dependent ratio of spiny dendrite capacitance load to proximal climbing fiber input is uneven in Purkinje cell dendrites, which causes inhomogeneous excitability of individual branches. In agreement with this theoretical study, the spatiotemporal variability of dendritic spikes has also been observed in awake mice by voltage imaging (Roome and Kuhn, 2018). Many factors can enhance the intrinsic inhomogeneous excitability *in vivo*, including firing state-related availability of K^+^ channels, concurrent synaptic input and compartment-specific dendritic excitability plasticity (Kitamura and Hausser, 2011; Ohtsuki et al., 2012; Zang et al., 2018). The significant spatio-temporal variability of dendritic Ca^2+^ influx implies that both LTP and LTD can occur coincidently at different dendritic branchlets of a Purkinje cell. This spatio-temporal variability can be fine-tuned to modulate the distribution of branchlet-specific synaptic changes, rather than a homogeneous change. Accordingly, the learning capacity of single Purkinje cells can be significantly increased, which may be necessary for complex and arbitrary movement learning (Bouvier et al., 2018).

## The Other Side of the Same Coin—Climbing Fibers as Motor Clocks

In contrast to the idea of climbing fibers as teachers in the Marr-Albus-Ito theory, an alternative view of their role is to provide a “motor clock” function in the initiation and timing of movements (Welsh et al., 1995; Llinás et al., 1997). Olivary neurons exhibit subthreshold oscillations, and neighboring neurons are coupled by gap junctions to help synchronize their outputs (Leznik and Llinás, 2005). Each climbing fiber also forms synapses on 5–10 Purkinje cells along the parasagittal direction. All these factors facilitate synchronization of complex spikes among neighboring Purkinje cells and spatio-temporally organize the output of Purkinje cell “microzones” (targeting the same cerebellar nucleus neuron). The ability to initiate or modulate movement seems to depend on synchronization of complex spikes in the Purkinje cell “microzone” rather than changes of complex spike firing rates (Llinás et al., 1997; Hoogland et al., 2015). Powerful climbing fiber synaptic current guarantees reliable conversion from synchronous climbing fiber input (“timing” signal) to synchronous complex spikes in Purkinje cells. First, climbing fibers evoke complex spikes immediately, which fits perfectly the high temporal precision required for the cerebellum to initiate or coordinate muscles. Second, although complex spike duration and spikelet number vary significantly under different conditions, the timing of the first several spikelets is relatively constant (Warnaar et al., 2015; Zang et al., 2018). This enables the timing and rate of spikes of cerebellar nuclei neurons efficiently entrained by synchronized presynaptic inputs from their upstream Purkinje cell “microzone” (Person and Raman, 2011). The view of climbing fibers as “motor clocks” is supported by some recent data. By simultaneously recording cerebellar nuclei neuron activities and complex spikes, Tang et al. (2019) observed inhibition of cerebellar nuclei neurons after complex spikes, with the degree of inhibition dependent on the synchrony among complex spikes. Delayed and attenuated co-activation of complex spikes have also been suggested to cause changes in the timing and execution of both complex and reflex movements (Hoogland et al., 2015).

**Figure 6:**
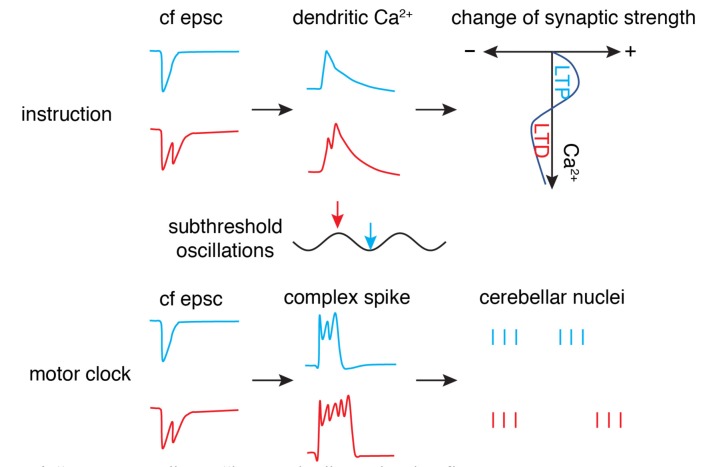
“Motor clock” and “instruction” by climbing fibers. The phase (timing) of synaptic input in subthreshold oscillations is encoded by the number of axonal spikes in a climbing fiber (cf), manifested by cf epsc. The burst can convert to dendritic spikes and modulate the Ca^2+^ influx to shift the polarity of synaptic changes (top panel) as an “instruction” signal. Simultaneously, the burst can convert to somatic complex spikes and entrain downstream cerebellar nuclei neuron outputs to initiate or modulate movement as a “motor clock” (bottom panel; Schematic, created by ourselves).

Is there any connection between the “motor clock” signal and instruction signal? The finding by Mathy et al. (2009) of climbing fiber bursting may well reconcile these two functions (Figure 6). The number of spikes in the climbing fiber burst depends on the phase of olivary subthreshold oscillations and can be read out by complex spike spikelet numbers and dendritic spikelet numbers in Purkinje cells. On one hand, the oscillatory phase of olivary neurons is converted to climbing fiber bursting and is then reliably transmitted to cerebellar nuclei neurons *via* complex spikes (Tang et al., 2019) to initiate or modulate movements. On the other hand, climbing fiber bursts can shift the probability of inducing short-term and long-term plasticity at parallel fiber synapses. However, this demonstrates what climbing fibers can do, rather than what they actually do. Further research is required to decipher whether the system is degenerate (Edelman and Gally, 2001), with both climbing fiber modes working simultaneously to improve motor performance, or whether the two modes are specific to particular cerebellar regions or distinct behaviors.

## Conclusions

Despite the growing awareness of multisite learning (Gao et al., 2012), climbing fiber-induced LTD and LTP in the cerebellar cortex may still comprise the backbone of cerebellar learning, especially now that climbing fibers have been demonstrated to be a versatile instructor. The ongoing activity-dependent analog instruction signal undoubtedly increases the learning capacity of the cerebellum.

## Author Contributions

YZ wrote the initial draft and revised it. ED edited the draft and revised it.

## Conflict of Interest

The authors declare that the research was conducted in the absence of any commercial or financial relationships that could be construed as a potential conflict of interest.
